# Amyloid β‐induced astrogliosis is mediated by β1‐integrin via NADPH oxidase 2 in Alzheimer's disease

**DOI:** 10.1111/acel.12521

**Published:** 2016-10-05

**Authors:** Ane Wyssenbach, Tania Quintela, Francisco Llavero, Jose L. Zugaza, Carlos Matute, Elena Alberdi

**Affiliations:** ^1^ Departamento de Neurociencias Universidad del País Vasco (UPV/EHU) 48940 Leioa Spain; ^2^ Centro de Investigación en Red de Enfermedades Neurodegenerativas (CIBERNED) Leioa Spain; ^3^ Achucarro Basque Center for Neuroscience 48940 Leioa Spain; ^4^ Departamento de Genética Antropología Física y Fisiología Animal Universidad del País Vasco (UPV/EHU) 48940 Leioa Spain; ^5^ IKERBASQUE Basque Foundation for Science María Díaz de Haro 3 48013 Bilbao Spain

**Keywords:** Alzheimer′s disease, amyloid‐β, astroglia, NOX2, β1‐integrin

## Abstract

Astrogliosis is a hallmark of Alzheimer′s disease (AD) and may constitute a primary pathogenic component of that disorder. Elucidation of signaling cascades inducing astrogliosis should help characterizing the function of astrocytes and identifying novel molecular targets to modulate AD progression. Here, we describe a novel mechanism by which soluble amyloid‐β modulates β1‐integrin activity and triggers NADPH oxidase (NOX)‐dependent astrogliosis *in vitro* and *in vivo*. Amyloid‐β oligomers activate a PI3K/classical PKC/Rac1/NOX pathway which is initiated by β1‐integrin in cultured astrocytes. This mechanism promotes β1‐integrin maturation, upregulation of NOX2 and of the glial fibrillary acidic protein (GFAP) in astrocytes *in vitro* and in hippocampal astrocytes *in vivo*. Notably, immunochemical analysis of the hippocampi of a triple‐transgenic AD mouse model shows increased levels of GFAP, NOX2, and β1‐integrin in reactive astrocytes which correlates with the amyloid β‐oligomer load. Finally, analysis of these proteins in postmortem frontal cortex from different stages of AD (II to V/VI) and matched controls confirmed elevated expression of NOX2 and β1‐integrin in that cortical region and specifically in reactive astrocytes, which was most prominent at advanced AD stages. Importantly, protein levels of NOX2 and β1‐integrin were significantly associated with increased amyloid‐β load in human samples. These data strongly suggest that astrogliosis in AD is caused by direct interaction of amyloid β oligomers with β1‐integrin which in turn leads to enhancing β1‐integrin and NOX2 activity via NOX‐dependent mechanisms. These observations may be relevant to AD pathophysiology.

## Introduction

Alzheimer's disease (AD) is a neurodegenerative disorder characterized by progressive loss of memory and cognitive function. The mechanisms underlying AD include an interplay between direct neurotoxicity, cerebrovascular pathology, inflammation, and cortical network dysfunction (Iadecola, 2004; Haass & Selkoe, 2007; Palop & Mucke, 2010). Whereas neuronal death has taken center stage in AD pathology, the putative contribution of glia to this disease has not been closely examined.

Astrocytes are the glial cells responsible for brain homeostasis and contribute to its protection in pathology (Kettenmann & Ransom, 2013). In neurological diseases, astrocytes show morpho‐functional changes to adopt a phenotype called astrogliosis that can affect the course of the disorder. AD patient brains have shown hypertrophic astrocytes clustering around amyloid plaques (Duffy *et al*., 1980), upregulation of astrogliotic marker GFAP in brain homogenates (Meda *et al*., 2001), and elevated levels of GFAP in the CSF of AD patients (Fukuyama *et al*., 2001). Overall, these data suggest a correlation between increased levels of these proteins in reactive astrocytes and disease severity.

Amyloid‐β (Aβ) species and their accumulation in plaques are key steps in the pathogenesis of AD (Haass & Selkoe, 2007). Astrocyte pathology might be a consequence of a primary Aβ‐induced glioreactivity over progression of the disease. Indeed, reactive astrocytes near Aβ plaques show impaired functions concerning Ca^2+^ network hyperactivity (Delekate *et al*., 2014) and an aberrant production of gliotransmitters (Jo *et al*., 2014) as well as disrupted astrocyte‐to‐astrocyte topology (Galea *et al*., 2015). In astrocytes, Aβ‐oligomers cause dysregulation of Ca^2+^ homeostasis (Abramov *et al*., 2003; Alberdi *et al*., 2013) and abnormal production of NADPH oxidase (NOX)‐derived reactive oxygen species (ROS) (Abeti *et al*., 2011) which leads to astrogliosis (Alberdi *et al*., 2013) and failure of astrocytic metabolism that may inflict neuronal cell death (Abramov *et al*., 2003; Abeti *et al*., 2011). Several Aβ‐oligomer receptors have been described, but no single candidate has been shown to be necessary and sufficient to account for all aspects of Aβ‐oligomer binding, glioreactivity, and toxicity (Viola & Klein, 2015). Among them, β1**‐**integrin was proposed as a partner in Aβ‐signaling (Sabo *et al*., 1995). Indeed, Aβ‐oligomers bind directly to low–intermediate activation conformers of β1**‐**integrin to induce mitochondrial and synaptic dysfunction in AD (Woo *et al*., 2015). In addition, Aβ‐peptide induces apoptosis and downregulation of α1β1 integrin in neuronal cells, indicating a relationship between Aβ‐neurotoxicity and modulation of integrin expression (Bozzo *et al*., 2004, 2010). Moreover, Aβ‐oligomers promote a high‐affinity state of LFA1 integrin to provoke a rapid neutrophil adhesion, trafficking and extravasation into the CNS in AD models which is a key to the progression of cognitive decline and gliosis (Zenaro *et al*., 2015).

In this study, we explored the mechanistic relationships among Aβ‐oligomers, β1‐integrin, NOX activities, and GFAP overexpression *in vitro*,* in vivo*, and in AD brain samples. We found that Aβ‐oligomers activate β1**‐**integrin/PI3K/PKC/Rac/NOX pathway to upregulate NOX2 expression and to promote β1‐integrin maturation, GFAP overexpression, and astrogliosis. We provide evidence showing that GFAP and NOX2 upregulation in Aβ‐injected mouse brain occurs through β1**‐**integrin signaling. Finally, analysis of a mouse model of AD and human AD samples shows that β1**‐**integrin and NOX2 levels are significantly higher in reactive astrocytes and correlate with Aβ‐load.

## Results

### Aβ‐Oligomers induce GFAP expression in response to NOX‐dependent redox regulation

**Figure 1:**
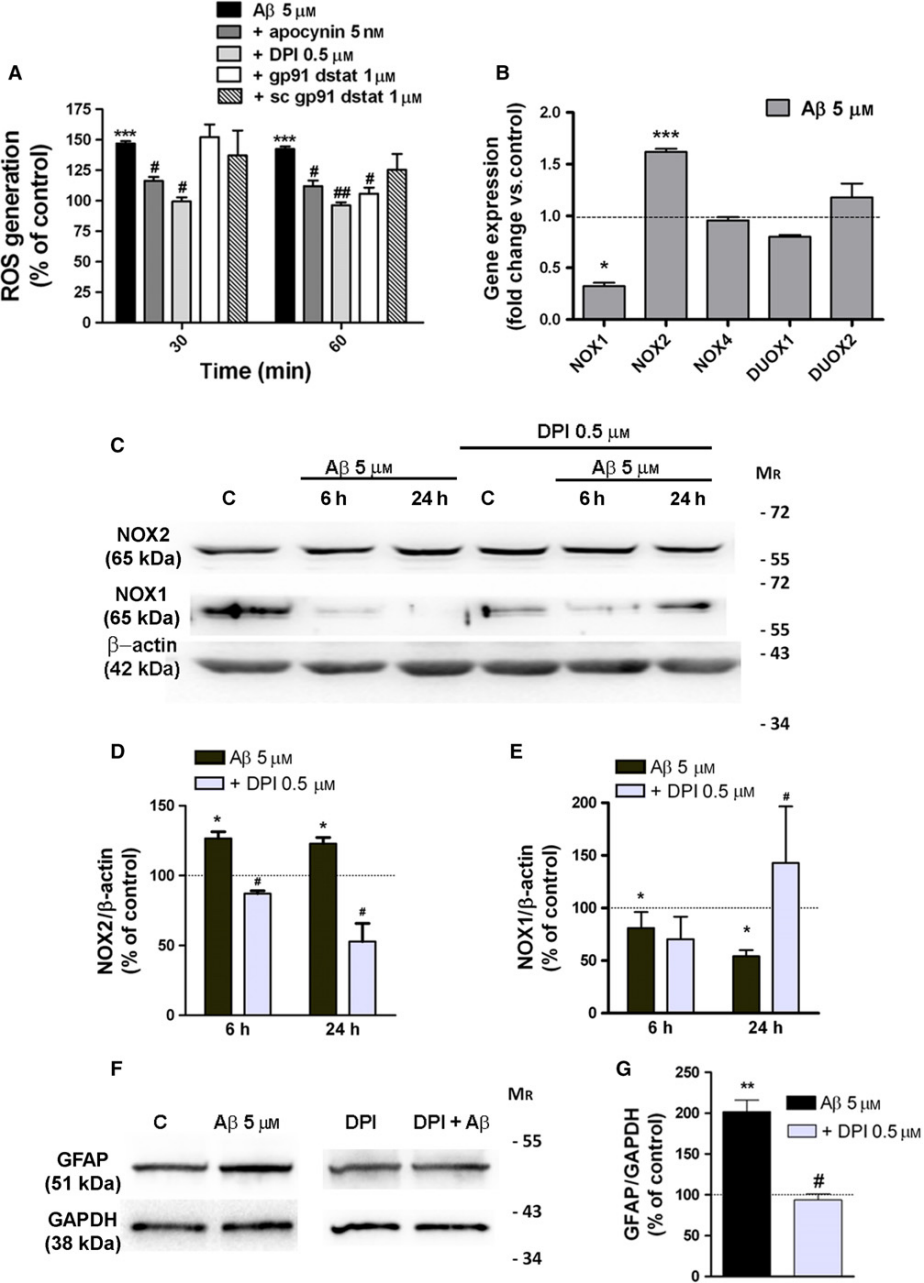
Aβ‐oligomers activate NOX and dysregulate its expression in cultured astrocytes. (A) Cells were treated with Aβ‐oligomers (5 μM, 30 and 60 min) or Aβ‐oligomers together with NOX inhibitors apocynin, DPI, gp91ds, and scramble gp91ds peptides. ROS generation was measured by fluorimetry with CM‐H2DCFDA (10 μM, 20 min). Data are expressed as relative fluorescence normalized to values of untreated cells or cells treated with inhibitors alone (100%). (B) Real‐time PCR was performed using specific primers for the target genes using RNA isolated from cultured astrocytes treated with Aβ oligomers. Bars represent fold change of gene expression normalized to values of untreated cells. Data represent the average of three independent cultures. (C–G) Astrocytes were treated with Aβ (5 μM) or with Aβ together with the NOX inhibitor DPI (5 μM), and protein expression (NOX1, NOX2, and GFAP) was analyzed by Western blot. Histograms represent the intensities of bands normalized to β‐actin, or GAPDH levels displayed as a percentage of the nontreated cells or DPI‐treated cells (100%). *P < 0.05, **P < 0.01, ***P < 0.001 compared to nontreated cells, ^#^ *P* < 0.05, ^##^ *P* < 0.01 compared to Aβ‐treated cells.

First, we found by real‐time fluorescence measurements that Aβ‐oligomers (5 μM) induced a rapid increase in fluorescence that reached plateau after 30–60 min (Fig 1A). This increase (142 ± 9%, *n* = 20 compared with untreated cells after 60 min) was blocked by compounds that prevent the assembly of NOX, apocynin (APO; 111 ± 13%, *n* = 8 compared to cells treated only with APO; Cavaliere *et al*., 2012), and DPI (96 ± 5%, *n* = 8 compared to cells treated only with DPI). In addition, astrocytes treated with a peptide that inhibits p47 (phox) association with NOX2 (gp91ds‐tat) showed a robust attenuated oligomeric Aβ‐induced signal (105 ± 9%, *n* = 4) (Fig 1A). We also analyzed the role of Aβ in the regulation of mRNA transcription and protein expression of NOX catalytic forms in cultured astrocytes. Here, we found that, among the NOX family, the mRNA levels of NOX2 were increased (1.6‐fold), whereas NOX1 decreased (0.3‐fold) (Fig 1B). Western blot analysis further confirmed the increase in NOX2 (129 ± 9% and 122 ± 9%, *n* = 6) and the decrease in NOX1 (55 ± 17% and 39 ± 9%, *n* = 6) protein levels after Aβ treatment for 6 and 24 h. DPI treatment abolished Aβ‐induced expression changes of NOX2 and NOX1 of astrocytes (Fig 1C–E). Overall, these data show that Aβ‐oligomers activate NOX enzymes and modulate NOX expression in a ROS‐dependent manner. In addition, we observed that GFAP overexpression in astrocytes treated with Aβ‐oligomers was reduced by NOX inhibition with DPI compound (Fig 1F,G). This result revealed that ROS production in astrocytes by Aβ‐oligomers could be a key signaling in the expression changes of GFAP levels.

### Aβ‐Oligomers activate classic PKC and Rac 1 GTPase to generate ROS

**Figure 2:**
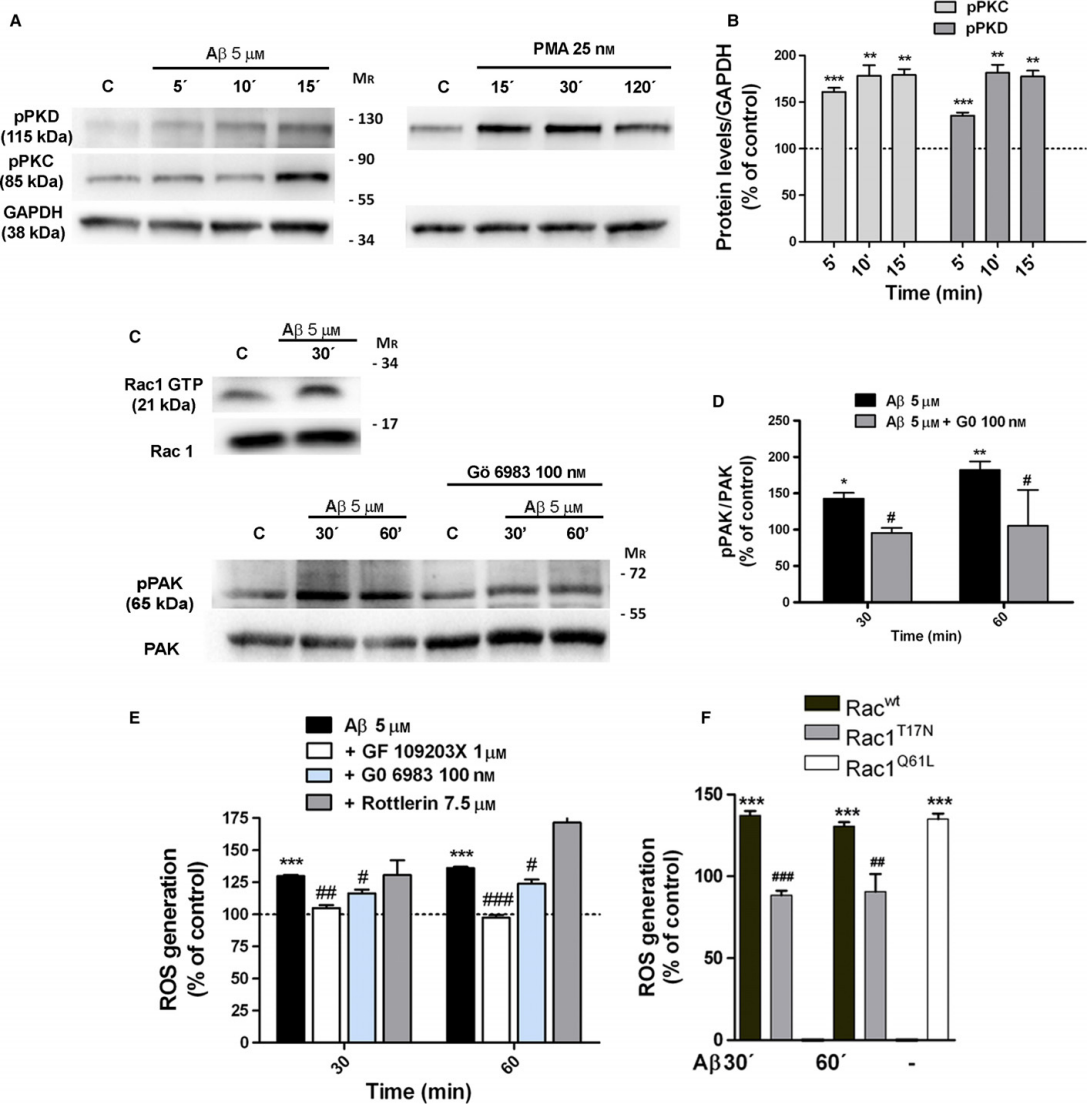
Aβ‐oligomers activate Rac1 through classical PKC activation. (A) Western blot analysis of the time course of PKC and PKD phosphorylation induced by Aβ‐oligomers (5 μM) and PMA (25 nM) in cultured astrocytes is shown. (B) Graph bars represent the intensities of bands normalized to GAPDH values displayed as a percentage of untreated cells as control. (C, left) Astrocytes were treated with Aβ‐oligomers (5 μM) for 30 min, and cell extracts were used to measure Rac1 activation (loaded with GTP) by affinity precipitation assay and PAK phosphorylation. Rac 1 and total PAK show total loading controls. (C right, D) Effect of Gö 6983 (100 nM) treatment in Aβ‐induced PAK phosphorylation as analyzed by Western blot. Graph bars represent the intensities of pPAK normalized to total PAK loading and expressed as a percentage of untreated cells or inhibitor‐treated cells as controls. (E) Differential effects of PKC inhibitors on Aβ‐induced ROS levels. Astrocytes were pretreated with PKC inhibitors GF109203X, Gö6983 and Rottlerin and subsequently with Aβ 5 μM. (F) Astrocytes were transfected with plasmids coding for Rac1^wt^, Rac1^T17N^, Rac1^Q61L^. In (E) and (F), ROS levels were measured in nontreated cells or cells treated with Aβ‐oligomers (5 μM, 30, 60 min). The results show the relative fluorescence normalized to untreated cells or PKC inhibitor‐treated cells (100%). *P < 0.05, **P < 0.01, ***P < 0.001 compared to nontreated cells; ^#^ *P* < 0.05, ^##^ *P* < 0.01, ^###^ *P* < 0.001 compared to Aβ‐treated cells or to Rac1^wt^ cells.

In many cell types, ROS production by NOX activity is mediated by PKC‐dependent activation of the small GTPase Rac, which is a component of the NOX enzyme complexes. As Aβ‐peptides elevate PKC activity in neurons (Manterola *et al*., 2013) and astrocytes (Abramov & Duchen, 2005), we analyzed whether PKC and Rac might function in a signaling axis to induce the NOX activity by Aβ‐oligomers in astrocytes. First, we verified that the PKC activator, phorbol 12‐myristate 13‐acetate (PMA 25 nM), produced a robust phosphorylation of PKD in astrocytes, because phosphorylation of PKD and PKC has been described as PKC activity markers (Manterola *et al*., 2013) (Fig 2A). Treatment of astrocytes with Aβ‐oligomers (5 μM; 5, 10 and 15 min) was able to induce a rapid and sustained phosphorylation of PKC (135 ± 10%; 181 ± 26%; 177 ± 24%, *n* = 13) and PKD (160 ± 15%; 178 ± 34%; 179 ± 25%; *n* = 13) (Fig 2A,B). Then, we examined whether ROS production mediated by Aβ‐oligomers involved PKC activation, using GF109203X (1 μM), a broad‐spectrum inhibitor for PKC family members, Gö 6983 (100 nM) and Rottlerin (7.5 μM), inhibitors of classic and novel members of PKC family, respectively. We found that GF 109203X and Gö 6983 reduced significantly the ROS levels after 30 and 60 min of Aβ‐treatment (109 ± 6%, 97 ± 4% and 116 ± 9% and 123 ± 10%, *n* = 11, respectively) as compared with the levels produced by Aβ‐treatment alone (128 ± 8% and 136 ± 8%, *n* = 11; Fig 2E). In addition, Rac1 activation analysis was performed using Rac1‐GTP pull‐down assays and quantification of phosphorylated levels of the Rac1‐dependent effector kinase, PAK1 (Fig 2C,D). Aβ‐Oligomers rose levels of Rac1‐GTP (154 ± 12%, *n* = 3) and phosphoPAK1 (130 ± 13%, *n* = 5) in astrocytes after 30 min postaddition (Fig 2C,D), compared to untreated cells as control (100%). This response was completely blocked by the co‐treatment of Aβ‐oligomers with PKC inhibitor Gö 6983 (95 ± 5% compared with cells treated only with inhibitor 100%, *n* = 5; Fig 2C,D).

To examine whether Rac1 activates NOX enzymes upon Aβ‐stimulation, we transfected astrocytes with expression constructs carrying full length of wild‐type Rac1, a dominant negative form of Rac1 (RacT17N) and a constitutively active form of Rac1 (Rac1Q61L). As shown in Fig 2F, Aβ increased ROS level in astrocytes expressing the wild‐type form of Rac1 (137 ± 2% and 130 ± 2%, *n* = 3), whereas failed in astrocytes expressing the dominant negative form of Rac1 (88 ± 2% and 90 ± 10%, *n* = 3). However, expression of RacQ61L resulted in a significant increase in ROS production (135 ± 2%) without Aβ‐stimulation compared to control cells (Fig 2F). These results suggest an active role of classical PKCs in Aβ‐induced Rac1 activation of NOX‐dependent ROS signaling in astrocytes.

### β1‐Integrin‐PI3K signaling is required for Aβ‐oligomer‐induced PKC and NOX activation

**Figure 3:**
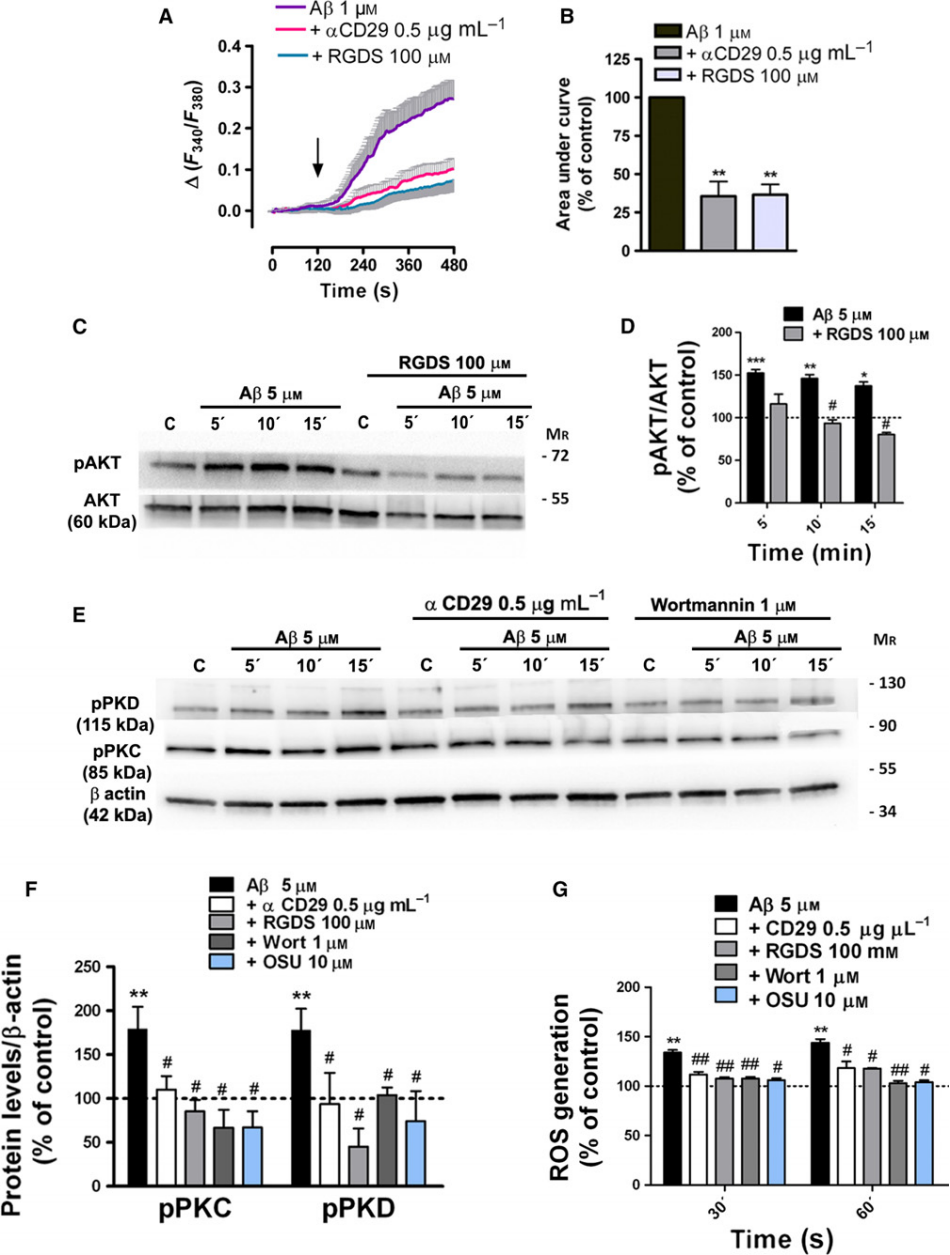
Aβ‐oligomers mobilize intracellular Ca^2+^ via β1‐integrin and activate β1‐integrin/PI3K signaling in astrocytes. (A, B) Ca^2+^ responses to Aβ 1 μM in astrocytes freshly isolated from hippocampal sections from P12 rats. Inhibitory effects of RGDS peptide (100 μM) and antibody αCD29 (0.5 μg ml^−1^) on astrocyte [Ca^2+^]_i_ following Aβ oligomer treatment (100%). (C, E) Levels of phosphorylated AKT, PKC, and PKD and total AKT and β‐actin were measured by Western blot analysis of astrocyte protein extracts untreated or treated with Aβ (5 μM, 5, 10 and 15 min), RGDS peptide (100 μM), αCD29 (0.5 μg ml^−1^), wortmannin (1 μM), and OSU‐03012 (10 μM). (D, F) Histograms represent the signal intensity of phosphorylated protein bands after normalization with intensities of total AKT or β‐actin proteins and expressed as a percentage of untreated cells or inhibitor‐treated cells as control values (100%). (G) Effect of β1**‐**integrin, PI3K, and PDK1 inhibitors on Aβ‐induced ROS levels on cultured astrocytes. *P < 0.05, **P < 0.01, ***P < 0.001 compared to nontreated cells; ^#^ *P* < 0.05, ^##^ *P* < 0.01 compared to Aβ‐treated cells.

Here, we investigated the putative involvement of β1**‐**integrin and PI3K in the molecular mechanisms triggered by Aβ to activate NOX in astrocytes. First, integrin‐mediated response to Aβ‐oligomers was examined in acutely isolated astrocytes from rat hippocampus. Fluorometric measurements of [Ca^2+^]_i_ showed that Aβ‐oligomers induced intracellular Ca^2+^ increases (100%, *n* = 20 cells obtained from 4 rats, Fig 3A,B) that were blocked by co‐incubation of Aβ with RGDS peptide (36.6 ± 6.7%, *n* = 24 cells obtained from 4 rats, Fig 3A,B), which contains the integrin binding sequence and inhibits its function (Wright & Meyer, 1985), and with an antibody (Mendrick & Kelly, 1993) that specifically binds to the 130‐kDa β1‐integrin chain (αCD29, 35.6 ± 9.6%, *n* = 20 cells obtained from 4 rats, Fig 3A,B). Moreover, in cultured astrocytes, AKT phosphorylation by Aβ‐oligomers was reduced by RGDS peptide, indicating that Aβ‐peptide activates PI3K and PDK1 through integrin activity modulation (Fig 3C,D). The contribution of β1‐integrin/PI3K/PDK1 activity to downstream PKC/NOX signaling was examined in Aβ‐treated astrocytes, together with αCD29 antibody, RGDS peptide, the PI3K inhibitor wortmannin, and the PDK1 inhibitor OSU03012. Western blot analysis of cell extracts and ROS measurements showed that phosphorylation of PKC/PKD and ROS levels was reduced by each specific inhibitor (Fig 3E,G). Thus, our data provide evidence that Aβ‐oligomers activate integrin‐associated signaling pathways that regulate NOX‐dependent redox signaling in astrocytes.

### Aβ‐Oligomers regulate β1‐integrin maturation and GFAP overexpression by controlling ROS generation

**Figure 4:**
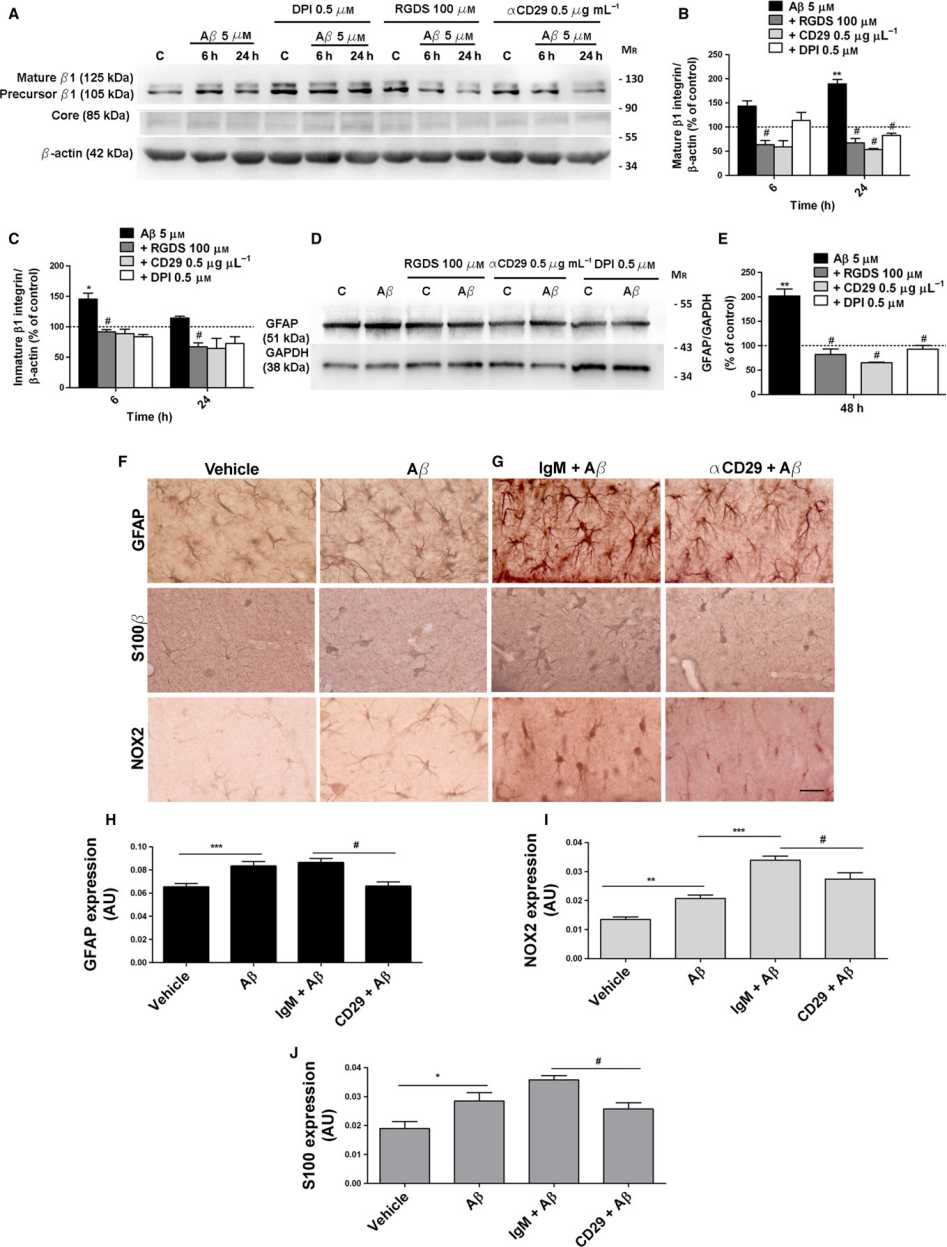
Aβ‐Oligomers promote β1‐integrin maturation and GFAP overexpression by activation of β1‐integrin‐NOX pathway *in vitro* and *in vivo*. (A) Astrocytes were treated with Aβ oligomers and with DPI, RGDS peptide, and αCD29. β1**‐**Integrin expression of astrocyte cell extracts was analyzed by Western blot and a 105‐kDa precursor form, a 125‐kDa mature form, and the core proteins (85 kDa) were noted in immunoblots. (B, C) Graphical bars show the mean intensities of mature form (B) and precursor form (C) of β1**‐**integrin normalized to β‐actin values. Data are expressed as a percentage of untreated cells or inhibitor‐treated cells as control values (100%). (D) Inhibitory effect of integrin inhibitors RGDS and αCD29 and the NOX inhibitor DPI on Aβ‐induced (48 h) GFAP overexpression. (E) Data represents levels of GFAP protein normalized with GAPDH values. (F, G) Coronal sections of mouse brains were analyzed after 7 days of injection with (F) vehicle and Aβ (125 ng) in the presence of (G) isotype IgM or αCD29 (1.35 μg) which blocks β1**‐**integrin. Photomicrographs showing GFAP, S100β, and NOX2 immunolabeling in astrocytes of the dentate gyrus are shown. Scale bar: 50 μm. (H, I, J) Bar graphs show the mean values of labeled areas for GFAP, S100β, and NOX2 under different conditions normalized to vehicle or (IgM+Aβ) values. Twelve slices per condition were analyzed. *P < 0.05, **P < 0.01, ***P < 0.001 compared with nontreated cells or control mice; ^#^ *P* < 0.05 compared to Aβ‐treated cells or IgM + Aβ‐injected mice.

Sequential glycosylation of β1**‐**integrin subunit (85 kDa) to form β1**‐**integrin precursor (105 kDa) and its mature form (125 kDa) represents a very important mechanism for its function modulation (Zheng *et al*., 1994). Next, we further investigated the role of Aβ‐oligomers in controlling the conversion of the β1**‐**integrin precursor to its mature form in astrocytes. Analysis revealed that, after 6 h of treatment, Aβ increased the levels of precursor β1 integrin (145 ± 24%, *n* = 6; Fig 4A,C), whereas 24 h of treatment increased the levels of the mature form (189 ± 22%, *n* = 4; Fig 4A,B). DPI inhibitor, RGDS peptide, and antibody αCD29 markedly decreased the levels of precursor and mature form levels (Fig 4A–C), suggesting that the intracellular ROS‐produced after binding of Aβ‐ to β1**‐**integrin may modulate functional levels of β1‐integrin in astrocytes. In addition, we observed that GFAP overexpression in astrocytes treated with Aβ‐oligomers was reduced by β1‐integrin activity inhibition with RGDS peptide and antibody αCD29 (Fig 4D,E).

Taken together, these data show that Aβ‐oligomers promote β1**‐**integrin maturation and GFAP overexpression by mechanisms that involve the Aβ‐binding to β1**‐**integrin receptors and the subsequent oxidative signaling in astrocytes.

### β1‐Integrin mediates the effects of soluble Aβ‐oligomers on hippocampal astrocytes *in vivo*

To extend the above characterization of biological effects of Aβ‐oligomers to *in vivo*, we injected Aβ‐oligomers (125 ng) or vehicle alone into hippocampus of C57 adult mice. Aβ‐injection led to astrocyte reactivity in the dentate gyrus compared with vehicle‐injected mice, as revealed by quantification of immunolabeled area with astrocyte markers GFAP (Fig 4F,H ANOVA, *P *<* *0.001; Tukey post hoc vehicle vs Aβ,*P *<* *0.001) and S100 β (Fig 4F,J ANOVA, *P *<* *0.001; Tukey post hoc vehicle vs Aβ, *P *<* *0.05) and with the oxidative stress marker NOX2 (Fig 4F,I ANOVA, *P *<* *0.001; Tukey post hoc vehicle vs Aβ, *P *<* *0.01). Co‐injection of Aβ‐oligomers with antibody against β1**‐**integrin (antibodies 1.35 μg) markedly diminished the GFAP (Fig 4G,H ANOVA, *P *<* *0.001; Tukey post hoc IgM+Aβ vs CD29 + Aβ, *P *<* *0.05), S100β (Fig 4G,J ANOVA, *P *<* *0.001; Tukey post hoc IgM+Aβ vs CD29 + Aβ, *P *<* *0.05), and NOX2 levels (Fig 4G,I, ANOVA, *P *<* *0.001; Tukey post hoc IgM+Aβ vs CD29 + Aβ, *P *<* *0.05) in astrocytes compared with them of isotype IgM‐injected mice (*n* = 6 animals in all instances). Unexpectedly, isotype IgM antibody enhanced significantly the NOX2 activation by Aβ compared with Aβ alone (Fig 4G,I ANOVA, *P *<* *0.001; Tukey post hoc IgM+Aβ vs Aβ, P < 0.001). Although the nature of this enhancement is unknown, it could be due to the presence of autoantibodies to astrocyte proteins including GFAP or oxidative stress markers (El‐Fawal & O'Callaghan, 2008).

Overall, these histological findings demonstrate that amyloid‐β oligomers trigger NOX2 upregulation and astrogliosis through β1**‐**integrin signaling in the mouse hippocampus.

### Oligomeric amyloid‐β levels correlate with β1‐integrin, GFAP, and NOX2 levels in 3xTg‐AD

**Figure 5:**
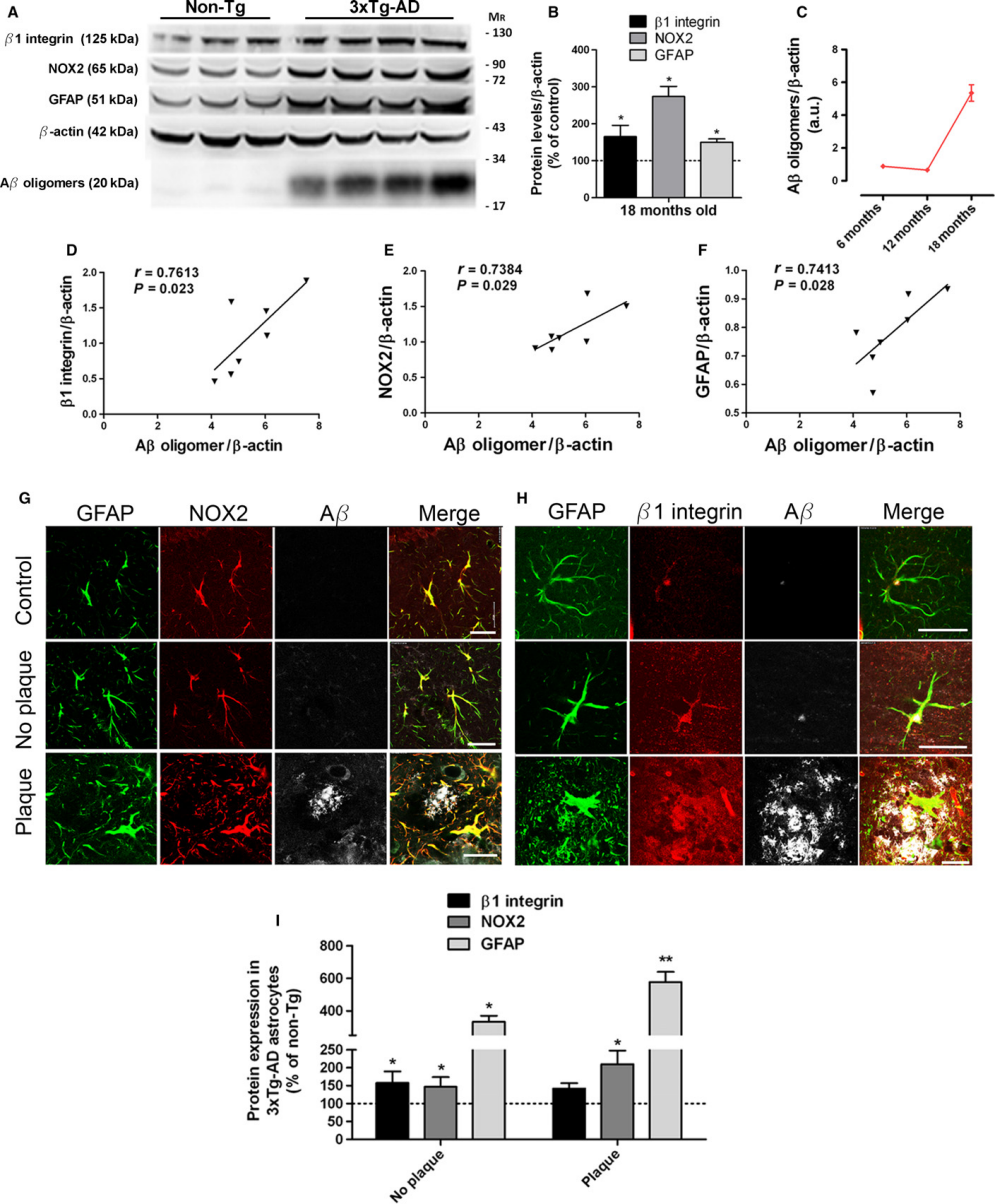
Increased levels of β1‐integrin, NOX2, GFAP in astrocytes of hippocampi of 3xTg‐AD (A) Representative images of Western blots of β1**‐**integrin, NOX2, GFAP, and oligomeric Aβ‐expression in hippocampi of 18‐month‐old 3xTg‐AD and non‐Tg mice. (B, C) Graphs showing relative protein levels to β‐actin (*n* = 16 mice). Data are expressed as a percentage of non‐Tg values (100%). (C) Protein levels of oligomeric Aβ in 3xTg‐AD mice were found to correlate with (D) β1**‐**integrin, (E) NOX2, and (F) GFAP in 18‐month‐old 3xTg‐AD mice. (G, H) Photomicrographs of triple immunofluorescence staining for GFAP (green), NOX2, and β1**‐**integrin (red) and Aβ (white) on coronal sections from non‐Tg and 3xTg‐AD mice. Scale bar: 20 μm. (H) Quantitative analysis was performed by measuring fluorescence intensity by pixel sum of β1**‐**integrin and NOX2 localized in GFAP^+^ cells. (I) Bars represent values in 3xTg‐AD mice relative to those obtained from non‐Tg mice. *P < 0.05, **P < 0.01, compared with non‐Tg mice.

We subsequently used the 3xTg‐AD mouse model (Oddo *et al*., 2003, 2006) to further assess changes in the expression levels of NOX2 as well as of β1**‐**integrin and their correlation with astrogliosis and the amyloid load. First, we detected detergent‐resistant amyloid‐β oligomers (pentamers of ~20 kDa) in hippocampal samples of 6, 12, and 18‐month‐old 3xTg‐AD animals, and levels were significantly increased in 18‐month‐old animals (Fig 5C). In addition, expression levels of β1**‐**integrin (165 ± 30%), NOX2 (273 ± 27%), and GFAP (150 ± 9%) increased significantly in 18‐month‐old 3xTg‐AD mice compared to age‐matched non‐Tg controls (100%; *n* = 8 animals in all instances) (Fig 5A,B). Notably, we found a robust correlation of levels of β1**‐**integrin (r = 0.7613, *P* = 0.023), NOX2 (r = 0.7384, *P* = 0.029), and GFAP (r = 0.7413, *P* = 0.028) with amyloid‐β oligomers in hippocampus (Fig 5D–F, respectively). Overall, these results indicate that overexpression of β1**‐**integrin, NOX2, and GFAP is associated with increased levels of amyloid‐β oligomer in aged 3xTg‐AD mice.

Next, we analyzed whether β1**‐**integrin and NOX2 levels were elevated in reactive astrocytes in the vicinity of amyloid plaques or apart from them. Quantification of confocal images, as pixel sum of each antibody labeling in GFAP‐positive pixels, showed an increased expression of these protein levels in hippocampal astrocytes of 18‐month‐old 3xTg‐AD mice (integrin β1: 158 ± 32% and NOX2: 147 ± 27%), as well as GFAP protein levels (334 ± 36%, *n* = 3), compared to age‐matched non‐Tg mice (100%). These increases were more pronounced in astrocytes surrounding the amyloid plaques (GFAP: 578 ± 62%, *n* = 3; β1**‐**integrin: 142 ± 15%, *n* = 3; NOX2: 210 ± 38%, *n* = 3) compared to age‐matched non‐Tg mice (100%) (Fig 5G–I). Taken together, these data show that astrocytes from the hippocampus of 3xTg‐AD mice expressed higher β1**‐**integrin and NOX2 levels compared to non‐Tg mice and suggest that the increased expression of these proteins correlates with the levels of soluble β‐amyloid peptide.

### Reactive astrocytes from prefrontal cortex of AD patients show higher levels of β1‐integrin and NOX2

**Figure 6:**
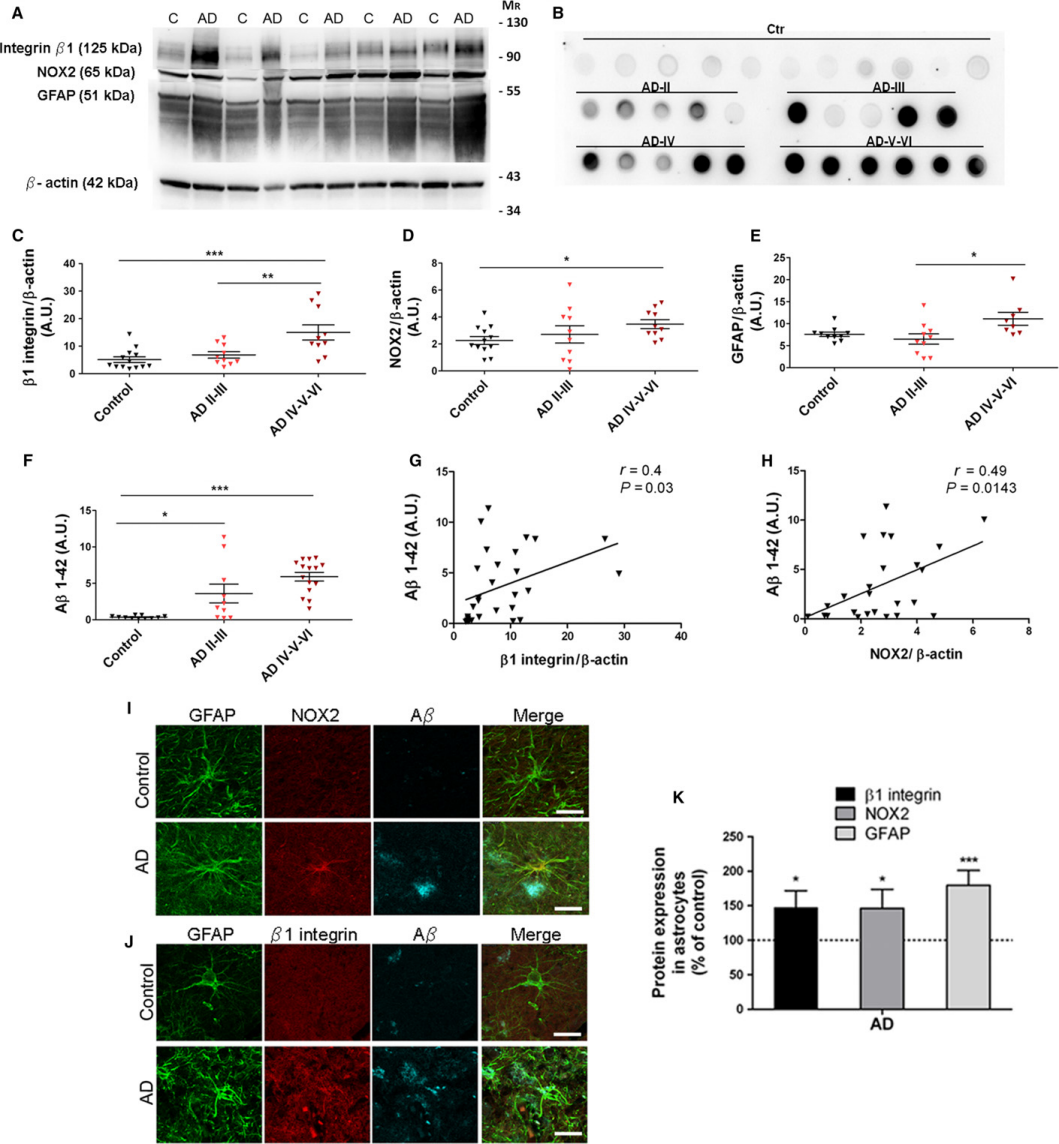
Increased expression of β1‐integrin, NOX2, and GFAP proteins in prefrontal cortex from AD patients. (A) Western blots show protein levels of β1**‐**integrin, NOX2, and GFAP in postmortem samples of prefrontal cortex from AD patients stage V‐VI (Braak and Braak) and nondemented controls. (B) Dot blot shows Aβ1‐42 protein levels in nondemented controls and AD patients classified into different Braak stages. (C–F) Plots illustrate protein levels as arbitrary units and normalized to β‐actin or GAPDH load, in control and AD samples of different stages of the disease. Protein levels of Aβ 1‐42 correlate with (G) β1**‐**integrin and (H) NOX2 in controls and AD patients. *P < 0.05, **P < 0.01, and ***P < 0.05 compared to nondemented control or AD‐II‐III. (I, J) Photomicrographs of triple immunofluorescence staining for GFAP (green), NOX2 and β1‐integrin (red), and Aβ (blue) on human prefrontral cortex samples from AD (Braak stage V‐VI) and matched controls. Scale bar: 20 μm. (K) Quantitative analysis of fluorescence intensity was performed by pixel sum of β1**‐**integrin and NOX2 localized in GFAP^+^ cells. Bars represent relative values obtained from control samples *P < 0.05, ***P < 0.001 compared to nondemented control.

In order to assess the relevance to AD of dysregulated levels of β1‐integrin and NOX2 in reactive astrocytes, we examined by Western blot, dot blot, and immunohistochemistry assays postmortem samples of prefrontal cortex brain from thirteen control and twenty patients with Alzheimer's disease classified as AD‐II, III, IV and V‐VI (Table 1) (Braak & Braak, 1995). Interestingly, the Western blot analysis revealed that β1‐integrin expression was upregulated during the progression of the disease (Fig 6A,C ANOVA, *P *<* *0.01; Tukey post hoc Control vs AD‐IV‐VI, *P *<* *0.001; Tukey post hoc ADII‐II vs AD‐IV‐VI, *P *<* *0.01). In addition, NOX2 and GFAP expression showed an increase in the latest stages (Fig 6D,E; ANOVA, *P *<* *0.005; Tukey post hoc Control vs ADIV‐VI, *P *<* *0.05; Tukey post hoc AD‐II‐III vs ADIV‐VI, *P *<* *0.05, respectively).

**Table 1:** Characteristics of human controls and AD patients, categorized as stages I to VI of Braak and Braak, used in this study

Case number	Braak stage		Gender	Age	Age (mean ± s.d.m)	Postmortem delay^a^
	NFT	Aβ				
C1	–	–	M	78		6:00
C2	–	–	M	83		13:00
C3	–	–	M	73	79.2 ± 1.3	6:10
C4	–	‐	M	76		11:30
C5	I	–	M	80		10:00
C6	–	–	F	86		4:00
C7	–	–	M	78		6:00
C8	–	–	F	69		12:00
C9	–	–	M	80		4:30
C10	–	–	M	79		4:45
C11	–	–	F	82		5:00
C12	–	–	M	80		5:30
C13	–	–	M	86		10:15
AD1	II	–	M	78		6:00
AD2	II	–	M	64		10:00
AD3	II	–	F	86	76.4 ± 3.6	13:30
AD4	II	A	F	75		16:20
AD5	II	–	F	79		10:30
AD6	III	A	M	73		4:20
AD7	III	–	M	84		15:00
AD8	III	–	M	74	77.8 ± 3.2	9:00
AD9	III	B	M	87		2:30
AD10	III	B	M	71		10:30
AD11	IV	B	M	82		2:30
AD12	IV	B	M	75		10:00
AD13	IV	B	M	70	79 ± 3.2	5:00
AD14	IV	B	M	79		5:30
AD15	IV	C	M	89		7:00
AD16	V	C	M	76		5:00
AD17	V	C	M	79		4:15
AD18	V	C	M	79	76.4 ± 0.6	5:00
AD19	VI	C	F	77		5:00
AD20	VI	C	M	77		5:00
AD21	VI	C	F	73		3:30
AD22	VI	C	F	74		3:30
AD23	VI	C	F	74		6:30
AD24	VI	C	M	78		7:00
AD25	VI	C	F	77		5:30

a *Time elapsed between death and sample extraction.

Dot blot assay showed an increase in Aβ1‐42 content during the progression of the disease (Fig 6B,F; ANOVA, *P *<* *0.001; Tukey post hoc Control vs AD‐II‐III, *P *<* *0.05; Tukey post hoc Control vs AD‐IV‐VI, *P *<* *0.001) that significantly correlated with the levels of β1**‐**integrin (r = 0.4, *P* = 0.03) and NOX2 (r = 0.49, *P* = 0.014) (Fig 6G,H, respectively) in the cortical areas of human samples.

Prefrontal cortex brain sections were also analyzed to study whether the aberrant increase in β1**‐**integrin and NOX2 levels was observed in reactive astrocytes clustered around amyloid plaques. Quantification of confocal images from three representative cases of AD patients and matched controls (AD‐V‐VI; *n* = 100 astrocytes) showed an increased expression of β1**‐**integrin (Fig 6J,K) and NOX2 levels (Fig 6I,K) (147 ± 24%, and 146 ± 27%, respectively), as well as GFAP protein levels (179 ± 21%), compared to controls (100%) (Fig 6I–K). Overall these data show that astrocytes from the cortex of AD patients express higher β1**‐**integrin and NOX2 levels compared to nondemented controls, suggesting a role for these proteins in the progression of Alzheimer's disease.

## Discussion

Astrogliosis is a neuropathologic hallmark in AD whose severity strongly correlates with the density of reactive astrocytes and the robust increase in GFAP in both brain and CSF (Muramori *et al*., 1998; Fukuyama *et al*., 2001). In the present study, we unveiled a new mechanistic pathway that drives astrogliosis in AD‐like pathology. Thus, our data indicate that amyloid‐β oligomers modulate integrin receptor activity in astrocytes which ultimately results in GFAP upregulation through NADPH oxidase‐mediated redox signaling (Fig S1, Supporting information). This idea is further supported by the presence of elevated expression of β1**‐**integrin and NOX2 which correlate with amyloid β load *in vivo* models of AD and in the disease proper.

The primary goal of the current study was to unmask the molecular players that contribute to the dysregulation of astrocyte physiology during AD progression. It is well known in these cells that Aβ promotes calcium imbalance by mechanisms that involve endoplasmic reticulum activation and stress (Alberdi *et al*., 2013), regulation of L‐type calcium channel expression (Daschil *et al*., 2015), and calcium‐sensing receptor activation (Chiarini *et al*., 2016). In addition to these activities, the glial cellular stress generated via NOX enzyme activities may contribute to leading glia‐mediated neurotoxicity in AD (Angelova & Abramov, 2014). However, the impact of NOX in signal transduction of astrocyte dysfunction has not been explored in detail in experimental paradigms relevant to AD disease. Here, we first observed that Aβ oligomers enhance NOX2 activity and its expression in a ROS‐dependent manner, which results in increased GFAP protein expression in astrocytes *in vitro*. Because GFAP induction is a key to astrocyte process extension and their thickening in reactive gliosis in AD (Yang & Wang, 2015), we studied the upstream mechanisms of NOX activation as to shedding light on the pathological remodeling of astroglia associated with AD progression. In particular, we explored the mechanistic relationships among β1**‐**integrin, NOX2, and GFAP expression because amyloid‐β peptides can bind β1**‐**integrin (Woo *et al*., 2015) and in this manner activate NOX enzymes (Moraes *et al*., 2015). Thus, in neurons, the interaction of amyloid‐β peptides with integrins changes cell‐adhesion capacity (Sabo *et al*., 1995) and leads to ROS production, mitochondrial dysfunction, and apoptosis (Woo *et al*., 2015). In addition, a direct interaction between amyloid‐β peptide and integrin occurs in inflammatory events in AD models, as the activation of LFA1 receptor in neutrophil accumulation on vascular inflammation (Zenaro *et al*., 2015). However, the contribution of integrin activity to astrogliosis mechanisms in AD has not been reported.

Various properties have been attributed to integrin activity in astrocytes, such as an important role in defining cellular properties of the blood brain barrier in the cerebral cortex (Venkatesan *et al*., 2015), and its ability to attenuate astrogliosis after spinal cord injury (Renault‐Mihara *et al*., 2011). In addition to those properties, we have demonstrated here that β1**‐**integrin activation by amyloid‐β oligomers in cultured astrocytes elevates cytosolic Ca^2+^ levels and triggers PI3K/PKC/Rac/NOX2 signaling which results in NOX2 upregulation, a GFAP level increase, and β1‐integrin maturation. These findings strongly suggest that soluble amyloid‐β can activate and regulate β1**‐**integrin availability to exacerbate intracellular signals leading to oxidative stress and astrogliosis.

The ability of integrins to bind to ligands is governed by integrin conformation, or activity, and this is an important route to the regulation of integrin function (Paul *et al*., 2015). In particular, amyloid‐β oligomers exhibit direct high‐affinity binding to β1‐integrin in neutrophils (Zenaro *et al*., 2015) and in neurons (Woo *et al*., 2015), inducing a rapid adhesion of both human and mouse neutrophils to endothelial ligands and conformational alteration and loss of surface β1‐integrin in neurons. In astrocytes, we observed that amyloid‐β oligomers increase PI3K, Rac, and NOX activities, and GFAP levels, which in all instances were reverted by a β1‐integrin function‐blocking antibody and RGDS peptide. In addition, the conversion of precursor into mature β1‐form was promoted by Aβ‐treatment, suggesting that, unlike in neurons, the cell surface levels and function of β1**‐**integrin in astrocytes is upregulated by amyloid‐β oligomers. Together, these findings support the idea that these peptides bind to β1**‐**integrin in the astrocyte plasma membrane and serve as a key initiator of astrogliosis in AD.

The effects observed in cultured astrocytes were validated *in vivo* in the adult hippocampus by means of microinjection of amyloid‐β oligomers allowing a dissection of early astrogliosis via NOX activation prior to frank neurodegeneration. Furthermore, Aβ‐oligomer‐induced astrogliosis was neutralized by an antibody blocking β1‐integrin function confirming the role of this cell‐adhesion receptor. Correlation of β1**‐**integrin expression to astrocyte reactivity has been previously reported in a mouse model of spontaneous seizures. Astrocyte hypertrophy and upregulation of GFAP and vimentin were observed in mice with a conditional deletion of β1**‐**integrin. Interestingly, this reactive gliosis appeared in the absence of cell death and blood–brain barrier disturbances (Robel *et al*., 2009), suggesting that alterations in β1‐integrin‐mediated signaling may hence be a primary mechanism implicated in eliciting specific aspects of reactive gliosis and confirm the results observed in the Aβ‐injected mouse model.

Several transgenic mouse reproducing hallmarks of AD pathology have been developed based on the ability of expressing APP, presenilin, and tau mutations. In the current study, the 3xTg‐AD was used because brains from these mice accumulate amyloid‐β oligomers in an age‐dependent manner (Oddo *et al*., 2006), as confirmed here by quantification of Aβ‐oligomers and immunohistochemistry in hippocampal samples of 6‐, 12‐ and 18‐month‐old mice. In addition, we observed a striking correlation between the levels of Aβ‐load in 3xTg‐AD and of astrogliosis as measured by GFAP levels, as well as of β1**‐**integrin and NOX2. Indeed, astrogliosis is more prominent near the amyloid plaques which suggest that β‐amyloid drives the change in astrocyte phenotype by activating β1**‐**integrin and NOX2 in 3xTg‐AD. Finally and in agreement with the findings in this AD model, higher levels of β1**‐**integrin, NOX2, and GFAP were found in samples of the prefrontal cortex and in reactive astrocytes at advanced stages of AD (IV–VI). Importantly, an increase in β1 integrin and NOX2 expression was associated with increased levels of amyloid beta peptide. In addition, immunohistochemical studies in the AD brains revealed prominent astroglia reaction surrounding amyloid β plaques. Overexpression of integrins on human astrocytes has been extensively described in neoplastic human brains. Specifically, β1 integrin dysfunction has been associated with mechanisms of cell migration and brain invasion of glioma and astrocytoma cells (Paulus *et al*., 1993); however, no data are available for the contribution of β1 integrin to astrocyte dysregulation in neurodegenerative diseases. Moreover, analysis of AD brains has demonstrated that both frontal and temporal regions exhibit a significant increase in NOX2 activity and in NOX2 cytosolic subunits expression throughout the disease progression, most notably in the more advanced stages of the disease. Unlike our results, the NOX activation in AD brains was exclusively attributed to microglial cells (Ansari and Scheff, 2011) and not to astrocytes or neurons. Further studies are needed to elucidate the function of β1 integrin and NOX2 in astrocytes of human brains.

In conclusion, we have found a new mechanism underlying astrogliosis in AD, which highlights astrocytes as a primary target of amyloid‐β via activation of β1**‐**integrin and redox signaling through NOX2. In turn, our results suggest that intermediaries in the signaling cascade described in this study can be promising protein biomarkers in AD progression. Finally, we propose that targeting the mechanisms of β‐amyloid‐driven and integrin‐dependent astrogliosis may render astrocytes functionally competent and possibly ameliorate the course of AD.

## Experimental procedures

### Animals

All experiments were conducted under the supervision and with the approval of our internal animal ethics committee (University of the Basque Country, UPV/EHU). Animals were handled in accordance with the European Communities Council Directive. All possible efforts were made to minimize animal suffering and the number of animals used.

### Astrocyte cell culture

Primary cultures of cerebral cortical astrocytes were prepared from P0–P2 Sprague Dawley rats as described previously (McCarthy & de Vellis, 1980). After 8 days, cells were plated onto PDL‐coated plates and maintained for 2 days. Culture medium was replaced with IMDM with 1% FBS 24 h before Aβ‐treatment.

### Measurement of ROS generation

Astrocytes (1x10^4^) were exposed to Aβ‐oligomers (Alberdi *et al*., 2013) alone or together with antagonists or inhibitors, as indicated. Cells were loaded with 10 μM CM‐H2DCFDA, and ROS levels were assayed as is previously described (Alberdi *et al*., 2013).

### Preparation of freshly isolated astrocytes

Rat brains (P8‐P11) were coronally cut into 300‐μm‐thick slices and maintained in ice‐cold aCSF containing 75 mM sucrose bubbled with carbogen (pH 7.4). Slices were incubated for 20 min in aCSF (pH 7.4) supplemented with 1 μM sulforhodamine 101. Acutely isolated cells were obtained from slices as described previously (Matthias *et al*., 2003). Cells were seeded into coverslips coated with PDL, and astrocytes were identified as red fluorescent labeled cells. Intracellular Ca^2+^ levels were determined using Fura2 according to the previously described method (Alberdi *et al*., 2013).

### Pull‐down assay

Rac1 GTPase was pulled down as described previously (Arrizabalaga *et al*., 2012). Precipitated proteins from 1×10^6^ astrocytes were eluted and analyzed by Western blotting with anti‐Rac1 (1:1000, Millipore Ibérica, Madrid, Spain).

### RT–PCR

Total RNA was isolated from cultured astrocytes (1×10^5^) using RNA mini‐prep kit (Agilent Technologies, Santa Clara, CA, USA). First‐strand cDNA synthesis was carried out with reverse transcriptase Superscript TMIII (Invitrogen, Barcelona, Spain) using random primers. Specific primers for NOX1‐NOX4 and DUOX1, DUOX2 were obtained from a previous study (Reinehr *et al*., 2007). Real‐time quantitative PCRs were carried out with 25 ng of reverse‐transcribed RNA and 300 nM of primers diluted in SYBRGreen PCR master mix reagent (Invitrogen, Barcelona, Spain). Relative levels of expression of target genes were calculated by means of a normalization factor, based on the geometric mean of multiple internal control genes and calculated by software.

### DNA transfections

Astrocytes were resuspended and electroporated using Amaxa Basic Glial Cell nucleofactor Kit (Lonza, Basel, Switzerland), containing 3 μg DNA. The constructs used were pCELF‐AU5‐Rac1 wt, pCELF‐AU5‐Rac1 Q61L, and pCELF‐Flag‐Rac1 T17N (Arrizabalaga *et al*., 2013).

### Intrahippocampal injections

Adult mice (3–4 months), anesthetized with ketamine hydrochloride (80 mg kg^−1^) and xylazine (10 mg kg^−1^), were injected stereotasically into the hippocampus at the following coordinates: −2.2 mm from Bregma, 1.5 mm lateral to the sagittal suture, and 2 mm from the pial surface. Mice were divided into four groups (*n* = 6 per group) and injected with 3 μl of vehicle (DMSO 17%, Ham's F‐12 83%), Aβ‐oligomers (125 ng), IgM (BD Biosciences, CA, USA) plus Aβ‐oligomers (1.35 μg and 125 ng, respectively), or anti‐CD29 antibody (BD Biosciences, CA, USA) plus Aβ‐oligomers (1.35 μg and 125 ng, respectively). After 7 days, mice were fixed and processed as described previously (Alberdi *et al*., 2013).

### Brain specimens

Formalin‐fixed paraffin‐embedded sections from the prefrontal cortex and frozen samples of 25 patients with AD as well as thirteen controls were obtained from the Neurological Tissue Bank Hospital Clínic‐IDIBAPS Biobank (Table 1). AD samples were grouped by Braak and Braak classification (Braak & Braak, 1995), into four groups: AD‐II, AD‐III, AD‐IV, AD‐V‐VI. All samples were matched by age and gender.

### Immunohistochemistry

Coronal vibratome sections (40 μm) of the dorsal hippocampus from mice (Oddo *et al*., 2003) were collected and processed for histochemistry. Formalin‐fixed paraffin‐embedded sections (10 μm) from the cortex of AD patients and controls were immersed in xylene followed by alcohol content solutions (100°, 96°, 90° and 70°) and PBS. Antigen retrieval with the Universal Buffer and the Antigen Retriever (Aptum, Southampton, UK) was performed before immunofluorescence histochemistry. Mouse monoclonal antibody against GFAP (1:1000, Millipore Ibérica, Madrid, Spain) and anti‐beta‐amyloid 1–16 (1:1000, 6E10 BioLegend, San Diego, CA, USA), rabbit polyclonal antibody against S100β (1:100, DakoCytomation, Barcelona, Spain) and NOX2 (1:1000, Novus Biologicals, Littleton, CO, USA), and rat monoclonal against β1‐integrin (1:100, Millipore Ibérica, Madrid, Spain) were used to label proteins in 3–6 sections per animal. Quantification of GFAP, S100β, and NOX2 in injected animals was performed as previously described (Alberdi *et al*., 2013). Fluorescence‐stained sections were observed and photographed with a Leica TCS SP8 confocal microscope, and the pixel sum of NOX2 and β1‐integrin in GFAP‐positive pixels were quantified with the LASAF software (Leica Microsystems, Mannheim, Germany).

### Western blot analysis

Tissues from 3xTg‐AD mice and AD patients were homogenized with a douncer and sonicated in 200 μl of RIPA with a protease inhibitor cocktail (Complete, Mini EDTA‐free tablets, Roche, Mannheim, Germany). Astrocyte (1×10^5^) and tissue homogenates were centrifuged at 4°C for 10 min at 12,000 × *g,* and protein content in the supernatant was quantified with the Bio‐Rad Protein Assay (Bio‐Rad, Hercules, CA, USA). Cell and tissue extracts (10 μg) were loaded into gels and blots were developed with rabbit polyclonal anti‐integrin β1, antiphosphoPAK, anti‐PAK, antiphosphoPKC, antiphosphoPKD, antiphosphoAKT, anti‐AKT (1:1000, Cell Signaling Technology, Beverly, MA, USA), rabbit polyclonal anti‐NOX2, anti‐NOX‐1 (1:1000, Novus Biologicals, Littleton, CO, USA), rabbit polyclonal anti‐β actin (1:5000, Sigma, St. Louis, MO, USA), mouse monoclonal anti‐GFAP, anti‐Rac1, anti‐GAPDH (1:2000, Millipore Ibérica, Madrid, Spain), and anti‐beta amyloid 1–16 (1:1000, 6E10 BioLegend, San Diego, CA, USA). For dot blot assay, human tissue extracts (2 μg) were spotted into a nitrocellulose membrane, and rabbit polyclonal anti‐Aβ1‐42 (1:10000, Abcam, Cambridge, UK) was used for chemiluminescence detection.

### Statistical analysis

One‐way analyses of variance followed by Tukey post hoc tests, one‐tailed Student's t‐tests, and the Pearson correlation coefficient were used unless otherwise indicated. All data are represented as mean±s.e.m. Statistical significance was set at *P* ≤ 0.05.

## Funding

This study was supported by CIBERNED and by grants from Ministerio de Economía y Competitividad (SAF2013‐45084‐R), Gobierno Vasco, Ikerbasque, and Universidad del País Vasco. A.W. held a fellowship from UPV/EHU and T.Q. from Gobierno Vasco.

## Author contributions

AW, CM, EA wrote the manuscript; all others received and approved the manuscript. AW, TQ, FL, JLZ, CM, and EA performed experiments. AW, CM, and EA analyzed data. CM and EA supervised the project.

## Conflict of interests

None declared.

## Supporting information


**Fig. S1** Model diagram of the signaling cascade activated by amyloid β oligomers in astrocytes.
**Fig. S2** Aβ oligomers promote GFAP, S100 and NOX2 overexpression via β1 integrin signaling in vivo.
