# Supplementary material for: Amyloid β‐induced astrogliosis is mediated by β1‐integrin via NADPH oxidase 2 in Alzheimer's disease
Source: Aging Cell. 2016 Oct 5;15(6):1140–52. doi: 10.1111/acel.12521 (PMC6398528; doi:10.1111/acel.12521)
Supplement: Supplementary file 1 — Fig. S1 Model diagram of the signaling cascade activated by amyloid β oligomers in astrocytes. Fig. S2 Aβ oligomers promote GFAP, S100 and NOX2 overexpression via β1 integrin signaling in vivo. [file ACEL-15-1140-s001.docx]

**Supporting information**

RT-PCR

Specific primers were designed with the PrimerExpress 2.0 software (Applied Biosystems, Madrid, Spain). Primer sequences were as follows: rat NOX1 forward 5’-TAC GAA GTG GCT GTA CTG GTTG-3’, and reverse 5’-CTC CCA AAG GAG GTT TTC TGT-3’; rat NOX2 forward 5’-GGT TCC AGT GCG TGT TGCT-3’, and reverse 5’-TCT TAT GGA AAG TAA GGT TCC TGT CC-3’; rat NOX3 forward 5’-AAT CAC AGA GTC TGC CTG GACT-3’, and reverse 5’-ATC CAG ACT TTC ATC CCA GTGT-3’; rat NOX4 forward 5’-GGA AGT CCA TTT GAG GAG TCAC-3’, and reverse 5’-TGG ATG TTC ACA AAG TCA GGTC-3’; rat DUOX1 forward 5’-CCT TTG CTG CTT CCC CTT AGT-3’, and reverse 5’-CGG TCT TGT CTC TGG AGC CTC-3’ and rat DUOX2 forward 5’-CCA TCC TAG TCA AGA TGG AAA AC-3’, and reverse 5’-ACA CAA TGG CCT GGA TG-3’.

Constitutive genes were also designed and the sequences were as follows: rat GAPDH forward 5’-GAA GGT CGG TGT CAA CGG ATTT-3’, and reverse 5’-CAA TGT CCA CTT TGT CAC AAG AGAA-3’, rat HPRT1 forward 5’-ATG GAC TGA TTA TGG ACA GGA CTGA-3’, and reverse 5’- ACA CAG AGG GCC ACA ATGT-3’, rat B2M forward 5’-CAC CGA GAC CGA TGT ATA TGC TT-3’, and reverse 5’- TTA CAT GTC TCG GTC CCA GG-3’, and rat CicA forward 5’-CAA AGT TCC AAA GAC AGC AGA AAA-3’, and reverse 5’-CCA CCC TGG CAC ATG AATC-3’.

**
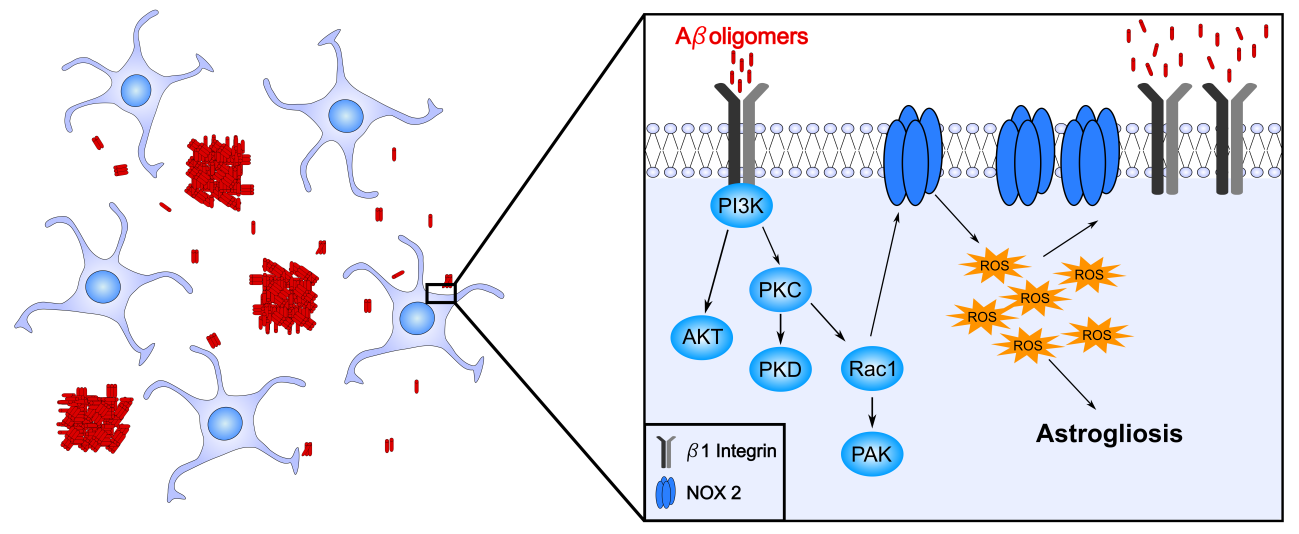
**

**Figure S1.** Model diagram of the signaling cascade activated by amyloid β oligomers in astrocytes. Briefly, toxic soluble amyloid β oligomers activate a PI3K/classical PKC/Rac1/NADPH oxidase pathway which is initiated by β1 integrin. This signaling mechanism promotes β1 integrin maturation as well as upregulation of NADPH oxidase 2 (NOX2) and the glial fibrillary acidic protein (GFAP) in cultured astrocytes and in hippocampal astrocytes *in vivo*

*
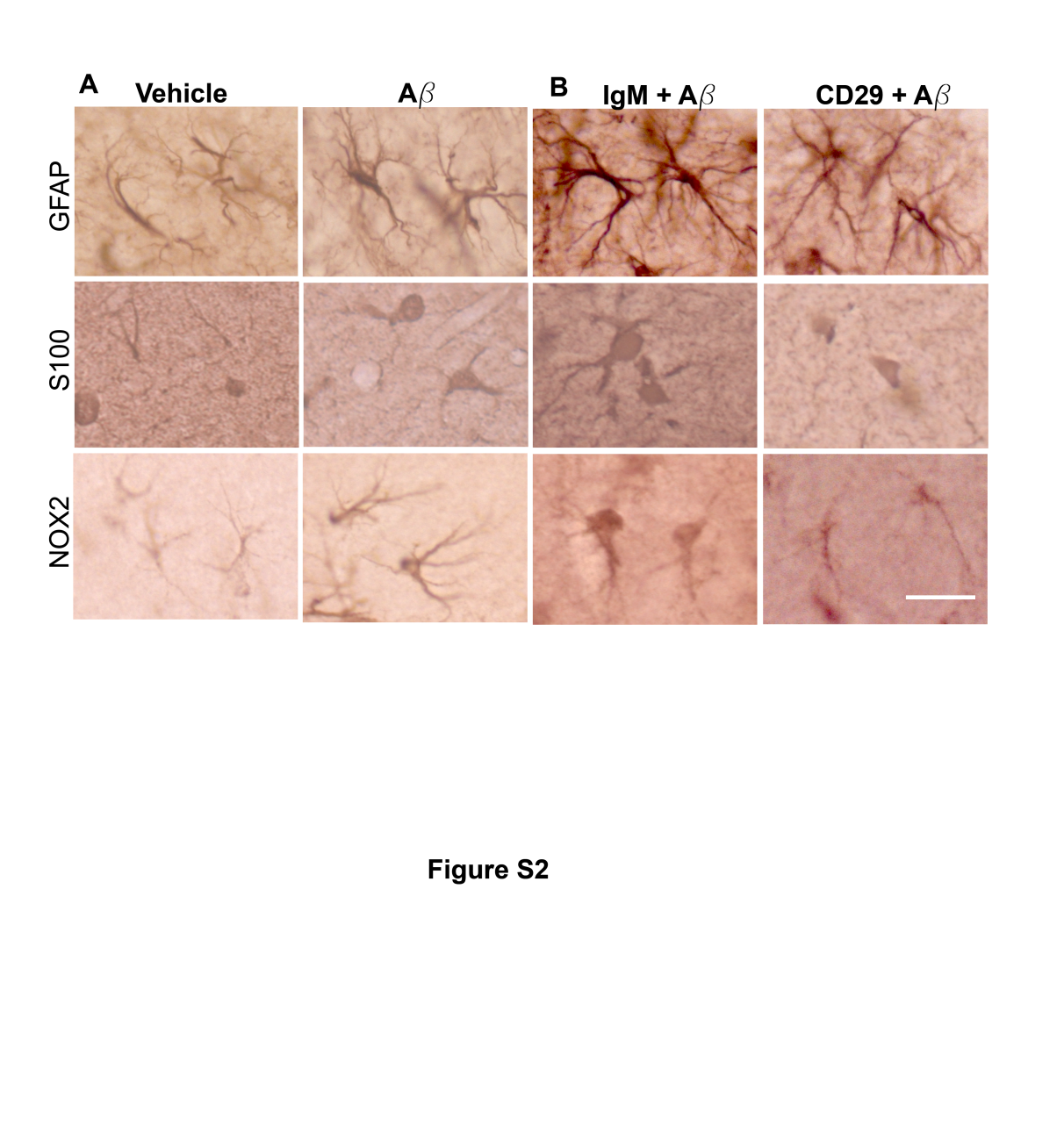
*

**Figure S2.** **Aβ oligomers promote GFAP, S100 and NOX2 overexpression via β1 integrin signaling in vivo.** Coronal sections of mouse brains after 7 days of injection with (A) vehicle and Aβ (125 ng) in the presence of (B) isotype IgM or αCD29 (1.35 μg) which blocks β1 integrin. Photomicrographs showing GFAP, S100 and NOX2 immunolabelling in astrocytes of the dentate gyrus are shown. Scale bar: 50 μm.
